# Comparison of transient elastography data among diabetic and non-diabetics: A study from teritiary health care centre in Kerala, India

**DOI:** 10.6026/973206300213218

**Published:** 2025-09-30

**Authors:** Rohith Kunnath, Satheesh A. Vasudevan, Sneha M. Nair, Rosemary Thomas, Sameena Sheeba Safarullah, Mark Anil Mansingh

**Affiliations:** 1 Department of Gastroenterology and Hepatology, P K Das Institute of Medical Sciences, Palakkad, Kerala, India; 2 Department of Forensic Medicine and Toxicology, Karnataka Medical College and Research Institute, Hubli, Karnataka, India; 3 Department of Community Medicine, P K Das Institute of Medical Sciences, Palakkad, India; 4 Department of Internal medicine, Al-Azhar Medical College and Super Speciality Hospital, Thodupuzha, Kerala, India; 5 Department of General Medicine, Locum Senior House Officer, Northern General Hospital, Sheffield, England

**Keywords:** Transient elastography, FibroScan, Diabetes mellitus (T2DM), non-diabetic population, Non-alcoholic fatty liver disease (NAFLD), Liver fibrosis, Liver stiffness measurement, Metabolic disorders, Hepatology, Fibrosis score comparison, Early detection, Risk stratification, Predictors of advanced fibrosis

## Abstract

Non-alcoholic fatty liver disease (NAFLD) is a common metabolic disorder with a strong association to type 2 diabetes mellitus (T2DM). Therefore, it is of interest to compare transient elastography findings among diabetic and non-diabetic individuals in a tertiary healthcare setting in Kerala. A total of 220 participants were assessed, of which 73 were diabetic and liver stiffness was measured using FibroScan®. Diabetic patients exhibited significantly higher mean fibrosis scores compared to non-diabetic patients (14.39 ± 14.42 vs. 8.14 ± 7.83, p=0.000). Thus, we show diabetes as a major predictor of advanced liver fibrosis, underscoring the need for early detection and targeted interventions in high-risk populations.

## Background:

Non-alcoholic fatty liver disease (NAFLD) is a serious chronic liver disease and an emerging international public health issue. In India, it is among the most common causes of cirrhosis, hepatocellular carcinoma (HCC) and liver transplantation [1]. Of especial note is the robust link between NAFLD and diabetes mellitus, diabetes being identified as an important driver of NAFLD progression. Prevalence of NAFLD in the Indian population is 9% to 53%, however, in high-risk groups like type 2 diabetes mellitus (T2DM), pre-diabetes, obesity and metabolic syndrome, the prevalence is much greater [2]. The actual burden of diabetes is high and increasing globally, with developing economies like India experiencing a particularly steep rise with respect to obesity and lifestyle changes. In India, the prevalence of diabetes grew from 7.1% in 2009 to 8.9% in 2019, affecting about 77 million people in 2019, a number that is expected to grow to 134 million by 2045. Scarily, about 57% of them go undetected [3, 4]. NAFLD consists of two forms: non-alcoholic fatty liver (NAFL), which is defined as ≥5% hepatic fat deposition without hepatocyte damage, and non-alcoholic steatohepatitis (NASH) with inflammation and hepatocyte ballooning with or without fibrosis. In a multicenter study done in 101 Indian cities, the incidence of NAFLD was 56.5% in 924 T2DM patients [5]. In addition to steatosis, the evolution to NASH and hepatic fibrosis points out the major role of NAFLD not only in liver disease but also in systemic complications [6]. The association between NAFLD and T2DM is established, with insulin resistance and compensatory hyper insulinemia inducing impaired lipid metabolism, hepatic triglyceride accumulation, and β-cell dysfunction [7]. T2DM patients are at increased risk for developing NAFLD and its more severe sequelae, such as fibrosis, cirrhosis and HCC [8]. In addition, NAFLD in T2DM patients enhances the risk of cardiovascular disease and diabetic vascular complications regardless of conventional risk factors [12]. Therefore, correct and precise estimation of NAFLD prevalence in T2DM patients is of utmost importance to forecast disease progression and apply timely interventions [9]. Our research was based in a tertiary care center in Kerala and targeted asymptomatic patients from the Palakkad district attending health camps conducted by PKDIMS and invited on social media. This community-based study is novel because sparse data exist on elastography scores in the general population and fibrosis evolution of diabetic patients in southern India. Therefore, it is of interest to characterize and compare transient elastography results between diabetics and non-diabetics in this community-based context.

## Methods:

Study population comprised of patients who came for transient elastography checkup in gastro medicine department. Medical history was taken from all individuals. Diabetic and non-diabetic cases were categorised separately. Height and weight were recorded. All patients went through targeted history and clinical examination to rule out liver disease. All patients underwent transient elastography, also known as FibroScan (®EchoSens), utilizing a 50-MHz transducer standard M-probe in our gastroenterology department. This non-invasive method involves ultrasound probes M and a vibrator. During the procedure, the patient lies on their back with their right hand fully extended, while a probe is positioned on the right lobe of the liver through an intercostal space on the body surface, guided by ultrasound. Vibrating waves are then directed to the specified liver tissue. To ensure reliability, at least 10 readings were taken from the liver area. A success rate of 60%, with an interquartile range of ≤30%, was established as the criteria for a valid measurement. The median FibroScan value displayed on the screen was recorded as the test result and used for subsequent analysis. Patients with known case of CLD/ Alcoholic liver disease were excluded. Results are displayed in tables and figures, with the categorical variables presented as numbers and percentages.

## Results:

The study included 220 participants, 73 being diabetic and 147 being non-diabetic. Most participants were male participants. The highest age category was 51 - 60 years, followed by the 41 - 50 years, and then the 61 - 70 years. Diabetic patients were significantly older in mean age compared to non-diabetic patients. BMI values were similar between groups. For diabetic patients, there were older age groups as compared to non-diabetic patients which had younger categories. Non-diabetic participants had more hypertension, but both groups had a high burden of comorbidities. FibroScan results showed that mean liver stiffness was statistically significantly higher for diabetic patients than non-diabetic patients indicating more advanced fibrosis. Due to the much greater mean liver stiffness for diabetic patients when we include other variables, we followed up further analyzing the associations of age and hypertension. In the analysis including the non-diabetic patients, increasing age and hypertension were associated with higher liver stiffness values; whereas year after year we do not see a statistically significant association with BMI in non-diabetic patients. Table 1 (see PDF) shows that diabetic patients had a higher mean age compared to non-diabetics (56.96 vs. 45.22 years). The BMI was similar across both groups, with slightly higher variability among non-diabetics. Table 2 (see PDF) demonstrates that the majority of diabetics belonged to the 61-70 years age group, whereas non-diabetics were mainly in the 31-40 years age group. Male predominance was observed in both groups. The majority of patients were overweight (BMI 25-29.9), and hypertension was more prevalent among non-diabetics. Table 3 (see PDF) outlines the FibroScan categories used to stage liver stiffness, ranging from F0-F1 (normal to mild fibrosis) to F4 (cirrhosis). Table 4 (see PDF) shows that diabetics had significantly higher mean FibroScan scores than non-diabetics (14.39 vs. 8.14, p=0.000), suggesting greater liver stiffness and fibrosis. Table 5 (see PDF) highlights the relationship between age, gender, BMI, and FibroScan scores. Among non-diabetics, age and hypertension showed a significant correlation with liver stiffness. No strong association was observed with BMI. Figure 1 (see PDF) illustrates the age-wise distribution of diabetic and non-diabetic participants. Diabetics were predominantly in the 61-70 years group, whereas non-diabetics were younger, concentrated in the 31-40 years range. Figure 2 (see PDF) compares the proportion of males and females in both groups. Males formed the majority in both groups, though the male-to-female ratio was slightly higher in non-diabetics. Figure 3 (see PDF) shows the BMI classification of participants across diabetic and non-diabetic groups. Overweight (BMI 25-29.9) was the most common category in both groups, followed by normal BMI. Figure 4 (see PDF) presents the proportion of participants with and without hypertension. Hypertension was more prevalent among non-diabetic patients compared to diabetics. Figure 5 (see PDF) demonstrates that diabetic patients had significantly higher FibroScan scores compared to non-diabetics, indicating more severe liver stiffness and fibrosis. Table 1 (see PDF) shows that diabetics were older on average compared to non-diabetics, while BMI values were similar across groups. Table 2 (see PDF) provides demographic details, showing that diabetics were concentrated in older age groups and that hypertension was more common among non-diabetics. Table 3 (see PDF) summarizes the classification of FibroScan scores, defining stages of fibrosis severity. Table 4 (see PDF) demonstrates a statistically significant difference in mean FibroScan scores, with diabetics having higher values than non-diabetics. Table 5 (see PDF) further explores associations between demographic factors and liver stiffness, where age and hypertension were significantly associated with fibrosis in non-diabetic patients, while BMI did not show significant correlations.

## Discussion:

Non-alcoholic fatty liver disease (NAFLD) is among the most common and leading global causes of chronic liver disease, associated with metabolic diseases, including diabetes mellitus. The results of this study show that diabetic patients had higher FibroScan scores than non-diabetic patients (14.39 ± 14.42 vs. 8.14 ± 7.83, p = 0.000) signifying higher liver stiffness and therefore more likely to have advanced fibrosis [10]. This corroborates the established assumption that diabetes hastens the progression of NAFLD to cause fibrosis, cirrhosis and hepatocellular carcinoma. A demographic analysis revealed that most diabetics were older and the majorities were male patients. Age and hypertension were significantly associated with higher FibroScan values in the non-diabetic patient population [11]. This demonstrates that liver fibrosis is also caused by metabolic and cardiovascular risk factors in the absence of diabetes. In terms of liver stiffness neither group demonstrated a significant association with BMI, which is in contrast to previous studies which identified obesity as a major contributor to NAFLD. These variabilities could be attributed to region, sample characteristics, or our small sample size [12]. The higher rates of fibrosis prevalent in diabetics can be ascribed to the effects of insulin resistance, disrupted lipid metabolism, and low-grade chronic inflammation, all of which have been noted in international studies further validating diabetes as a meaningful risk factor for progressive liver disease. Through comparison to previous studies done in Australia and India on a global scale, it is noted that liver fibrosis measures are more severe in diabetics (and other high-risk populations) and that daily use of transient elastography is justified in this population. All in all, the study stresses the important of diabetes is not only a metabolic disease but also a significant risk factor for the progression of chronic liver disease. Adding FibroScan to routine assessment in diabetics may allow for early detection of fibrosis and management strategies to mitigate future complications.

## Conclusion:

Diabetic patients have higher mean FibroScan scores, indicating severe liver fibrosis than non-diabetics. Age and hypertension are significant predictors of liver stiffness in non-diabetic individuals. Routine FibroScan assessment and multidisciplinary management can aid early diagnosis, lifestyle interventions, and mitigation of liver disease progression.
